# BisCEET: A Visual Browser for Biosynthetic Gene Clusters Aiding in the Identification of Natural Product Variants and Distinct Tailoring Enzymes

**DOI:** 10.1021/acs.jnatprod.5c01563

**Published:** 2026-04-29

**Authors:** Sven T. Sowa, Heiner G. Weddeling, Robin Teufel

**Affiliations:** Pharmaceutical Biology, Department of Pharmaceutical Sciences, 27209 University of Basel, Klingelbergstrasse 50, 4056 Basel, Switzerland

## Abstract

Genes involved in the biosynthesis of microbial natural products (NPs) are typically arranged in biosynthetic gene clusters (BGCs). Different congeners of an NP family typically possess distinct chemical features introduced by additional tailoring enzymes encoded in the corresponding BGC variants. However, tools to rapidly visualize the core gene set and distinguish it from variant-specific tailoring genes (VSTGs) in these BGCs are lacking. Here, the software tool BisCEET (Biosynthetic Cluster Environment Examination Tool) was developed, allowing comparison and visualization of the gene composition of related BGCs, thereby streamlining the identification of VSTGs in uncharacterized BGC variants and strains likely to produce novel NP congeners. The use of BisCEET is exemplified by analyzing bacterial BGCs of staurosporine-like indolocarbazoles and xantholipin-like polyketides, which enabled the identification of numerous apparent BGC variants. We anticipate that BisCEET will become a valuable bioinformatic asset, streamlining the prioritization of BGCs and the cultivation of microbial strains for the discovery of distinct NP variants and novel tailoring enzymes.

## Graphical abstract

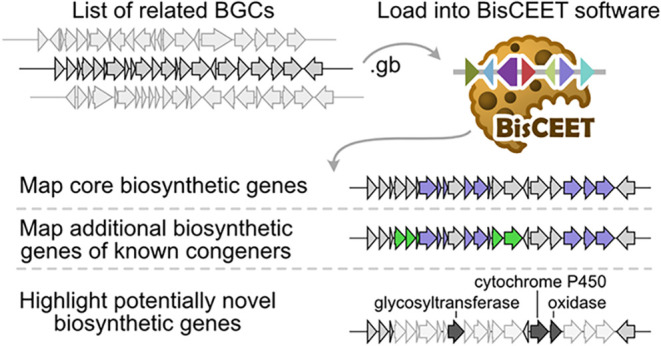

In bacterial and fungal genomes, genes encoding pathways for the production of NPs are often tightly clustered. These BGCs provide the benefit of coregulated gene expression and furthermore facilitate the rapid spread of functionally connected groups of genes through horizontal gene transfer.^1^ The resulting distribution of a given BGC then provides numerous possibilities to evolve in the context of the ecological niche occupied by these microbial species. For example, the diversification of BGCs and thus the emergence of BGC variants in the context of NP families can arise by complementing the “core biosynthetic genes” (CBGs; encoding enzymes predominantly involved in the early biosynthetic steps and formation of the NP backbone, but also including tailoring enzymes that generate shared features of all congeners of a certain NP family^2,3^ ) through acquisition of VSTGs coding for rare tailoring enzymes that further chemically alter and diversify the NP core scaffold and thus enable the production of distinct congeners.^4^ The modifications introduced by these tailoring enzymes comprise a wide array of reactions such as alkylation, glycosylation, oxygenation, or halogenation.^5–7^

In order to identify new BGC variants, VSTGs associated with BGCs of known NP types can therefore serve as a hallmark indicator. While excellent genome mining tools for automated classification of BGC families and acquisition of related BGCs exist,^8–15^ a standalone tool for the rapid comparison of related BGCs and visualization of potentially novel VSTGs could streamline genome mining for producers of novel NP congeners, for example, in the context of finding new antibiotics to combat antimicrobial resistance. In this work, we developed the tool BisCEET (**Bi**o**s**ynthetic **C**luster **E**nvironment **E**xamination **T**ool) for visual representation of BGCs and rapid identification of potentially novel VSTGs. We furthermore demonstrate how BisCEET can be practically applied in genome mining efforts by identifying potentially novel BGC variants of staurosporine-like indolocarbazoles and (heavily modified) pentangular polyketides.

## Results and Discussion

The genome mining-based search for novel NP congeners often begins with the identification of BGC variants that harbor distinct or uncharacterized biosynthetic genes. While existing bioinformatic pipelines, such as BIG-SCAPE,^12^ CORASON,^10^ GATOR-GC,^15^ and zol/fai,^14^ excel at large-scale orthology inference, they often lack the granularity required to spot subtle genetic variations (such as additional encoded tailoring enzymes) in closely related BGCs. BisCEET addresses this by functioning as a post acquisition “workbench” implemented as a dynamic visualization engine in a graphical user interface that prioritizes user interaction.

We differentiated the BisCEET workflow from that of standard phylogenomic tools through two key implementations:

1. Dynamic reanchoring: Unlike the static, predefined anchor points used in other visualization software such as CORASON, BisCEET allows users to interactively redefine CBGs. This instantly reorganizes the visual alignment, enabling the detection of variants that might be missed in rigid alignments.
2. Visual filtering: We developed an “Inspection View” to visually suppress conserved CBGs and optionally also genes annotated to encode proteins with no direct biosynthetic involvement such as transporters or regulators. This visual filtering specifically highlights potential VSTGs–coding for, e.g., oxidoreductases or glycosyltransferases–that are often key to identifying novel natural product congeners.

Consequently, BisCEET acts as a distinct interactive layer that integrates with and enhances the output of acquisition tools such as CluSeek or cblaster.

To translate these design principles into a practical workspace, BisCEET streamlines the comparison process into three distinct stages: data input, mapping CBGs (and known VSTGs), and visual inspection (Figures 1 and S1).

**Figure 1:**
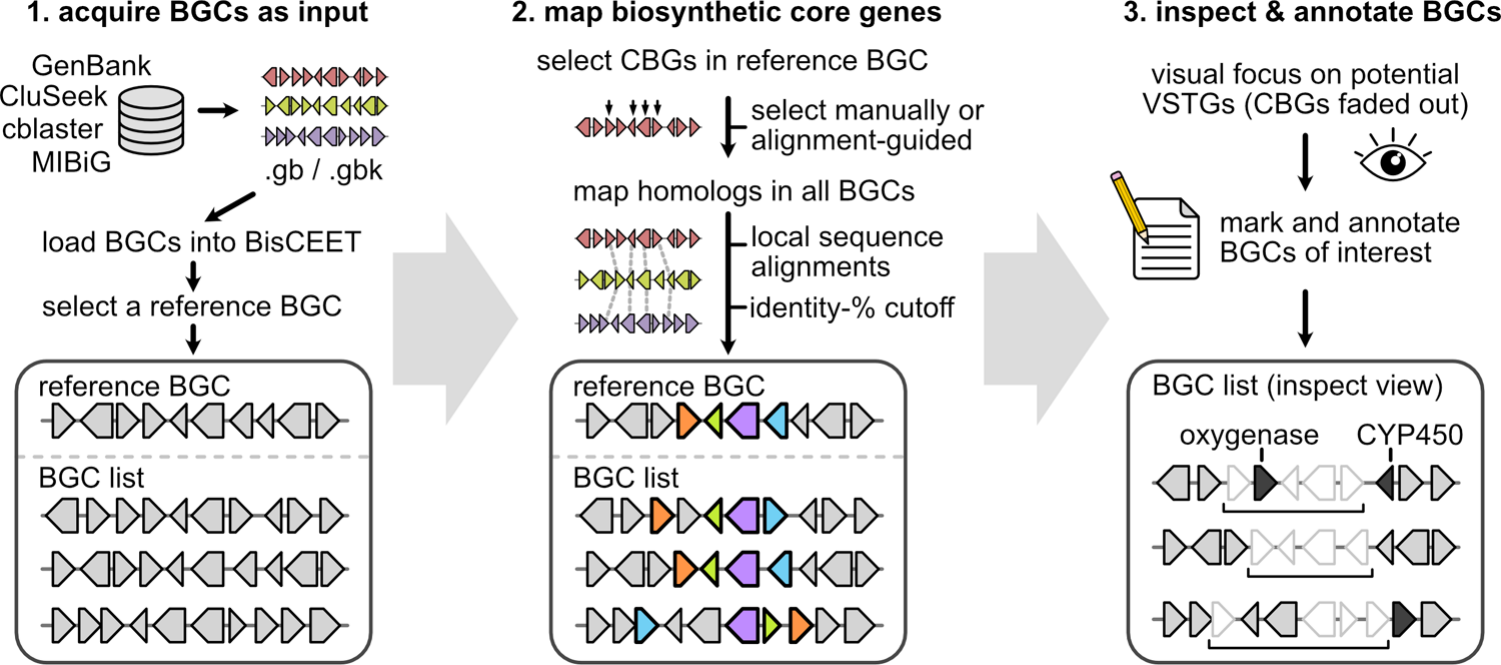
Workflow for the comparison and visualization of BGCs with BisCEET. The boxes are a schematic representation of the graphical interface in BisCEET.

For the initial input in BisCEET, BGCs of structurally related NPs are required in GenBank file format, which can be sourced directly from repositories such as NCBI GenBank, MIBiG, and the AntiSMASH database.^8,16^ Moreover, tools like CAGECAT (cblaster), MultiGeneBlast, and CluSeek can be used for automated acquisition of comprehensive sets of related BGCs.^9,11,17^ Once the BGCs are loaded into BisCEET, a single BGC is designated by the user as reference, which serves as a visual anchor (Figure S2) and should ideally be well-characterized. In order to identify CBGs, the user may either (i) select a number of CBGs based on previous studies and literature or (ii) allow BisCEET to automatically select core genes based on sequence alignments with genes from the other BGCs. To this end, the user can mark the desired number of BGCs from the list before BisCEET automatically selects the cBGs that have homologues in these clusters with a user-defined sequence-identity cutoff (default: 40%) based on pairwise local sequence alignments. Aside from CBGs selected in the reference BGC, VSTGs can be defined by the user (e.g., encoding distinct tailoring enzymes of known congeners). Next, homologues of any of the designated genes are identified in the list of BGCs and automatically colored according to the reference including selected additional genes (Figure S3).

For closer examination of BGCs and to facilitate identification of potential VSTGs, the BGCs can be visualized via the “inspector view” function. Here, the region comprising all mapped genes (CBGs and known VSTGs) is visually indicated for each BGC (Figure S4). The mapped genes themselves can be visually suppressed to highlight putative VSTGs present within or in the direct vicinity of the BGCs. Furthermore, the corresponding protein sequences and GenBank identifiers of the genes of interest can be retrieved from the interface, allowing their direct input in other bioinformatic tools, e.g., to identify the protein family of a putative VSTG. Moreover, the annotations derived from the corresponding GenBank file for each BGC are displayed directly above the nonmapped genes, superseding the need for manual selection and examination of each gene. This also simplifies the identification of groups of related genes, e.g., VSTGs encoding enzymes involved in the biosynthesis and conjugation of a sugar moiety, which typically cluster together. Genes whose annotations contain specific terms that imply biosynthetic relevance (e.g., “transferase”, “cytochrome P450”, “monooxygenase”, etc.) can additionally be highlighted in bold. Likewise, annotations for genes related to non-biosynthetic functions (e.g., “transporter”, “symporter”, “regulator”) can be hidden from view. Thereby, BisCEET enables the rapid identification of exactly those BGCs with putative novel VSTGs of interest (that likely enable the biosynthesis of uncharacterized NP congeners), which can be annotated and saved in BisCEET for further downstream analyses.

As a proof of concept, we performed the BisCEET workflow for the BGC variants of the rubromycin/griseorhodin family of aromatic polyketides (derived from a pentangular backbone^18–24^ ) that led to the discovery of a previously undescribed glycosylated griseorhodin congener, which we named ruskamycin (copublished in this issue: 10.1021/acs.jnatprod.6c00190).^37^ Below, two further examples are detailed to showcase the BisCEET workflow and identify BGC variants of indolocarbazoles and pentangular aromatic polyketides without further experimental verification of the putative novel NP congeners.

Indolocarbazoles are biosynthetically derived from two L-tryptophan moieties and often display diverse antiproliferative activity against cancer cells.^25^ Staurosporine-like indolocarbazoles are produced by bacteria and characterized by the presence of a sugar moiety that is attached via two C–N linkages (Figure 2A), distinguishing this class from similar indolocarbazoles that possess only a single C–N linked sugar (e.g., rebeccamycin)^26^ or lack sugar residues entirely (e.g., arcyriaflavins).^27^ For the initial C–N linkage, a dTDP-sugar is transferred by the *N*-glycosyltransferase StaG; the corresponding reaction in rebeccamycin biosynthesis is catalyzed by RebG.^28,29^ For staurosporine-like indolocarbazoles, the second C–N linkage between the glycoside and the second indole moiety is introduced by the distinct cytochrome P450 enzyme StaN.^30^ Only two BGCs of this class, enabling the biosynthesis of staurosporine and K-252a, were reported to date (Figure 2A), while many congeners were isolated and structurally characterized without knowledge of the corresponding BGCs (Figure 2B).^28^ The identification of such BGCs via homology searches for known CBGs would be straightforward; however, the species originally reported to produce these congeners had not been sequenced. Consequently, we sought to identify some of the corresponding staurosporine-related BGC variants among published bacterial genomes whose VSTGs and encoded tailoring enzymes would match the distinct structural features of the respective congener or, alternatively, find BGC variants predicted to enable the biosynthesis of previously unreported staurosporine-like NPs.

**Figure 2:**
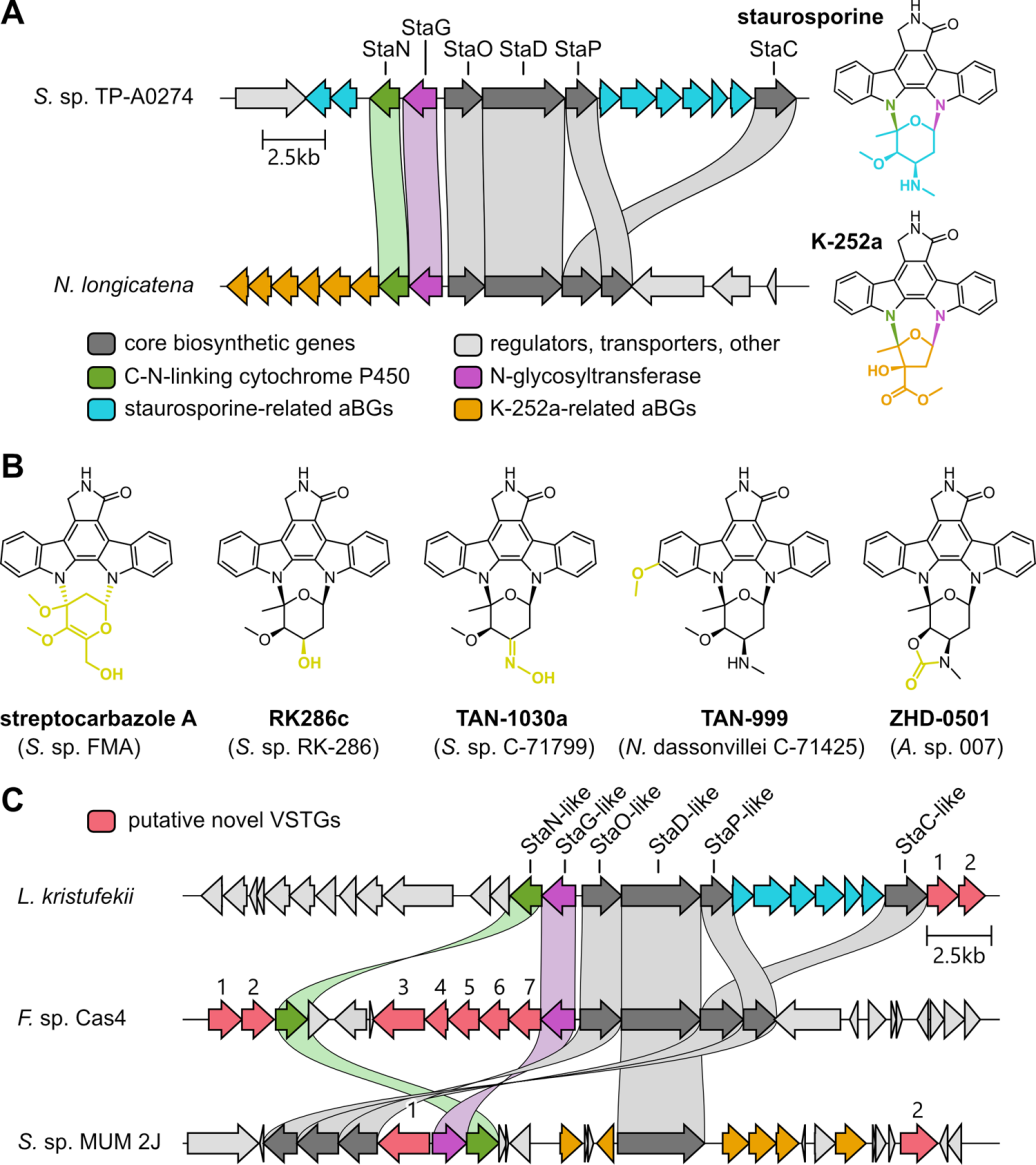
Genome mining for BGCs indolocarbazoles with cyclic N-glycosidic linkages. (A) BGCs of staurosporine and K-252a. (B) Examples of previously isolated indolocarbazoles with cyclic N-glycosidic linkages for which no BGCs were identified yet: Streptocarbazole A from *Streptomyces* sp. FMA; RK286c from *Streptomyces* sp. RK-286; TAN-1030a from *Streptomyces* sp. C-71799; TAN-999 from *Nocardiopsis dassonvillei* *C*-71425; ZHD-0501 from *Actinomadura* sp. 007. Structural features distinguishing these congeners from staurosporine are highlighted in yellow. (C) Novel BGC variants of interest identified with BisCEET. Putative novel VSTGs (red) are numbered and corresponding annotations are listed in the Supporting Information (Tables S1–S3). Connecting ribbons are shown between homologues of CBGs sharing over 40% sequence identity. The color scheme of the genes is according to the legend in panel (A).

As the first step, the genomic sequences of the co-localizing homologues of StaD and StaO (indolocarbazole CBGs) as well as StaG and StaN (glycosidic C–N linkages) within 60 kbp were retrieved from GenBank using CluSeek, resulting in a total of *N* = 196 BGCs. Next, the GenBank files of the staurosporine BGC from *Streptomyces* sp. TP-A0274 and the K-252a BGC from *Nonomuraea longicatena* were retrieved from MIBiG (accession numbers BGC0000825 and BGC0000814) and opened in BisCEET. The staurosporine BGC was set as a reference, and the K-252a BGC was marked as a favorite. Next, all remaining BGCs were opened in BisCEET. All CBGs responsible for the biosynthesis of the indolocarbazole scaffold StaO, StaD, StaP, and StaC as well as the C–N linking StaG and StaN were manually selected in the reference cluster.^31^ Genes responsible for the biosynthesis of the glycoside moieties from staurosporine and those of K-252a were added to the “gene bench” and selected. All selected genes were then mapped to the entire list of BGCs using the “map to gene clusters” function, and biosynthetically relevant genes among the BGCs were scrutinized in “inspection view”. Several BGCs of interest were identified (Figure 2C).

A BGC highly similar to the BGC of staurosporine was found in the genome of *Lentzea kristufekii*, which is directly flanked by genes encoding a putative cytochrome P450 as well as a methyltransferase (Table S1). These potential VSTGs would fit the biosynthetic logic required for the formation of the congener TAN-999 (Figure 2B), which contains an additional methoxy group in its indolocarbazole scaffold and could be installed by a hydroxylation (cytochrome P450) followed by methylation (methyltransferase), although experimental studies would be needed to confirm this. Another BGC of interest was found in *Frankia* sp. Cas4, which encodes four additional cytochrome P450 enzymes and three methyltransferases (Table S2) that could be involved in extensive tailoring of the scaffold. While this cluster does not contain genes associated with dTDP-sugar formation, homologues of StaG and StaN are present, which are responsible for installment of the cyclic N-glycosidic linkages; it is thus possible that the biosynthetic genes for dTDP-sugar biosynthesis are encoded elsewhere in the genome. Finally, a cluster from *Streptomyces* sp. MUM 2J harbors homologues for all genes required for biosynthesis of K-252a and additionally two genes predicted to encode an FAD-dependent monooxygenase and an FAD-dependent oxidoreductase, which may introduce further redox modifications to a K-252a-like scaffold.

As a second case example, we employed BisCEET to identify potential new congeners of pentangular polyketides xantholipin and the closely related lysolipin. Pentangular polyketides are structurally diverse polycyclic aromatic compounds produced by Actinobacteria and typically derived from a dodeca- or tridecaketide chain, often harboring many modifications introduced by extensive tailoring and ring-rearrangements.^19,21–23,32^ Xantholipin and lysolipin form a subgroup within pentangular polyketides, possessing a xanthone moiety (rings A–C), a chlorinated ring A, and a methylenedioxy group forming ring G as characteristic features introduced by corresponding tailoring enzymes (Figure 3A).^33^ In many pentangular polyketides such as hexaricin A (Figure 3B), rings A–C form an anthraquinone, while in xantholipin and lysolipin, the anthraquinone is transformed to a xanthone by the FAD-dependent monooxygenase XanO4.^34^ Chlorination of ring A is introduced by FAD-dependent halogenase XanH while the methylenedioxy group of ring G is formed by cytochrome P450 enzyme XanO2.^33,35^ Xanthone pentangular polyketides are widespread with over 50 congeners reported, however, the three structural features noted above are thus far only reported for xantholipin and lysolipin and similarly also for chloroalbofungin (Figure 3B) and its closely related congeners Sch 54445 and actinoplanone, which are halogenated at ring E or F instead of ring A and additionally feature a rare hydrazine moiety in Ring F.^36^ To collect BGCs likely to enable the biosynthesis of close congeners of xantholipin/lysolipin, genomic sequences with colocalized gene homologues encoding the aforementioned XanO2, XanH, and XanO4 as well as the polyketide synthase type II α subunit XanF within 60 kbp were retrieved from GenBank with CluSeek, resulting in a total of *N* = 193 BGCs. The GenBank files of xantholipin from *Streptomyces flaveogriseus* and lysolipin from *Streptomyces tendae* were retrieved from MIBiG (accession numbers BGC0000279 and BGC0000242) and loaded into BisCEET. The xantholipin BGC was set as a reference, and the lysolipin BGC was marked as a favorite. Next, all remaining BGCs were opened in BisCEET. To automatically identify CBGs among these large clusters, the lysolipin BGC was “marked” and the “select homologues from marked clusters” was executed, thus selecting all genes in the reference xantholipin BGC that have homologues in the marked lysolipin BGC with at least 40% sequence identity. Next, xantholipin- and lysolipin-related VSTGs were added to the “gene bench” and selected there. All selected genes were then mapped to the entire list of BGCs using the “map to gene clusters” function with a 40% sequence-identity cutoff. The “inspection view” was used to scrutinize each BGC in the list, making it possible to identify putative VSTGs within or in the vicinity of these BGCs.

**Figure 3:**
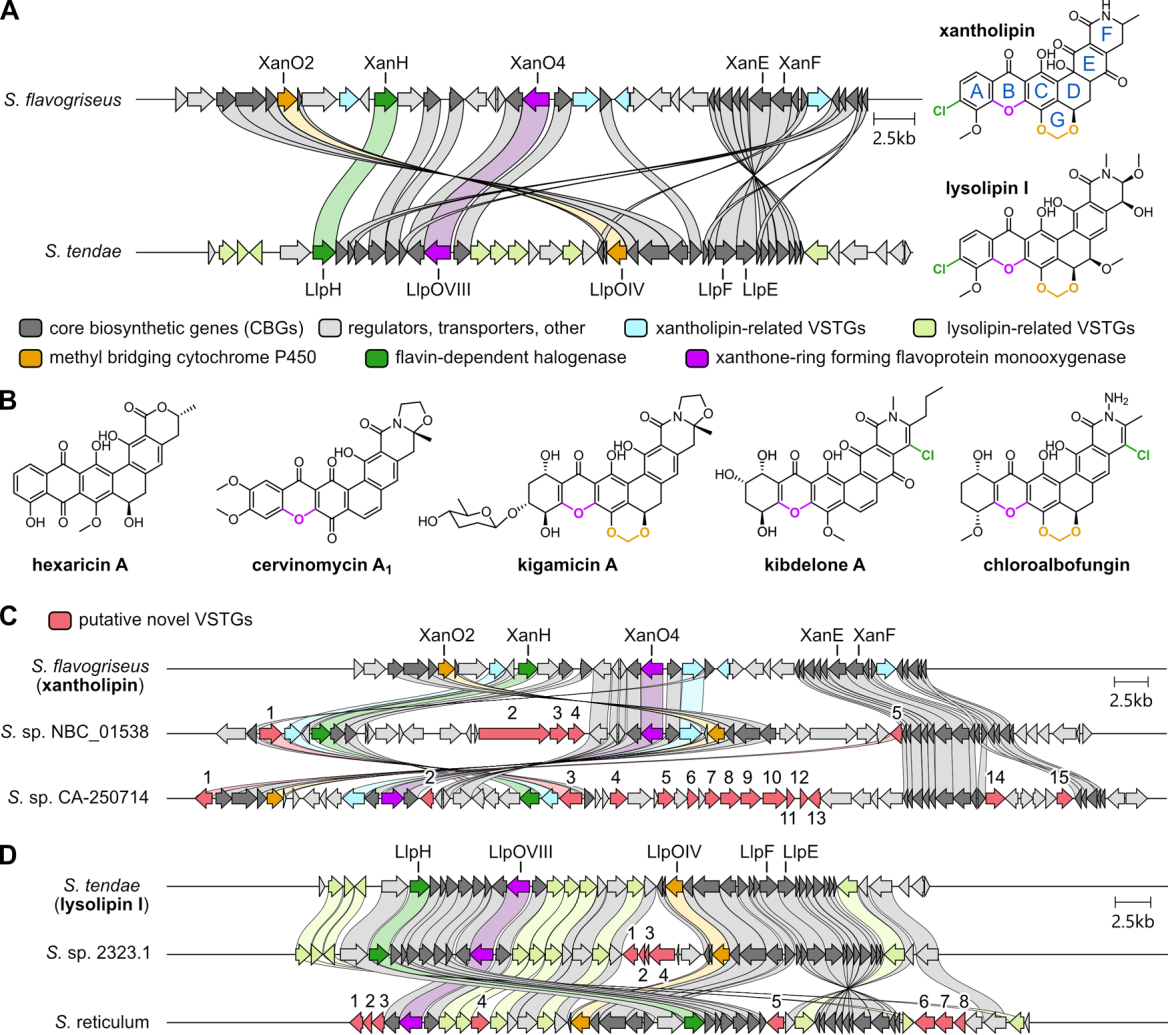
Genome mining for novel xantholipin- and lysolipin-like BGCs. (A) BGCs of xantholipin and lysolipin. Labels for genes encoding tailoring enzymes XanO2, XanH, and XanO4 as well as the type II polyketide synthase subunits XanE and XanF are shown. (B) Examples of pentangular polyketides. Structural features shared with xantholipin/lysolipin are highlighted in colors. Putative novel BGC variants of (C) xantholipin and (D) lysolipin identified in BisCEET. Putative novel VSTGs (red) are numbered, and corresponding annotations are listed in the Supporting Information (Tables S4–S7). Connecting ribbons are shown between homologues sharing over 40% sequence identity. The color scheme of the genes is according to the legend in panel (A).

Among the BGCs, several promising variants were found. In *Streptomyces* sp. NBC_01538, a BGC variant containing all selected CBGs and some xantholipin-related VSTGs was found, which in addition encodes two predicted oxidoreductases, a cytochrome P450 enzyme, and an NRPS-like protein (Figure 3C and Table S4). Another BGC of interest was identified in *Streptomyces* sp. CA-250714, where, in addition to the CBGs and known VSTGs, 15 genes with biosynthetically relevant annotations were found (Figure 3C and Table S5). Among these are genes annotated to encode oxidoreductases, methyltransferases, a cytochrome P450, a type III polyketide synthase, and aminotransferases. These enzymes could therefore be involved in substantial modifications of the xantholipin scaffold and potential biosynthesis of a hybrid NP, although it is also conceivable that these genes belong to a second, unrelated BGC, which remains to be investigated. In addition, two examples of putative BGC variants more closely related to lysolipin were found (Figure 3D). In *Streptomyces* sp. 2323.1, this BGC is identical in composition and synteny to the lysolipin BGC; however, it also contains a region comprising genes predicted to encode for protein-L-isoaspartate O-methyltransferase, transglutaminase, and asparagine synthase (Table S6). In *Streptoverticillium reticulum* strain J-35, this BGC closely resembles that of lysolipin but strikingly also contains genes related to the biosynthesis of an NDP-sugar moiety (Table S7) along with two glycosyltransferases and two methyltransferases and may therefore enable the formation of a glycosylated lysolipin congener.

This example illustrates the undemanding application of BisCEET even for highly complex BGCs withover 30 genes, including numerous CBGs and VSTGs, owing to the functionalities of hiding CBGs and known VSTGs in BisCEET while simultaneously highlighting potentially novel, biosynthetically relevant VSTGs.

## Conclusion

When analyzing and comparing hundreds of BGCs in genome mining efforts, researchers have to keep track of complex information, such as the identities of known core CBGs and possible VSTGs in the context of the genomic environment. BisCEET streamlines this process by converting static genomic data into a dynamic workbench, enabling the rapid and straightforward identification of potentially novel VSTGs and, thus, BGC variants. We illustrated that this workflow effectively identifies BGC variants likely responsible for producing novel NP congeners. BisCEET therefore enables researchers to quickly and effortlessly transition from large genome data sets to specific, high-value experimental targets.

## Experimental Section

### General Experimental Procedures

BGCs of reported compounds were obtained from the MiBIG BGC repository.^16^ CluSeek 2.0.2 ^11^ was used to collect BGCs from genomic sequences deposited in NCBI GenBank. Figures of comparisons between related BGCs were generated with clinker as part of the CAGECAT package.^9^

### Development of BisCEET

BisCEET version 1.0.0 is implemented in Python (version 3.12.3). It employs the Tkinter package from the Python standard library for the graphical user interface and also uses the third-party module Biopython (version 1.86) for sequence alignments. Sequence alignments are done pairwise in local mode using a BLOSUM62 substitution matrix and −20 open gap score as well as −5 extend gap score. Genes are visually mapped in BisCEET if they are above a sequence-identity threshold defined by the user (default: 40%), and if the alignment has a query coverage of over 50%. Biopython was obtained via Python Package Index.

### Identification of Novel BGC Variants

For retrieving staurosporine-like indolocarbazole BGCs, protein sequences of StaD (GenBank accession BAC55211.1), StaO (GenBank accession BAC55210.1), StaG (GenBank accession BAC55209.1), and StaN (GenBank accession BAC55208.1) were used as input for BLAST search in CluSeek. For retrieving xantholipin/lysolipin-like BGCs, protein sequences of XanO2 (GenBank accession ADE22287.1), XanH (GenBank accession ADE22292.1), XanO4 (GenBank accession ADE22300.1), and XanF (GenBank accession ADE22315.1) were used as input for BLAST search in CluSeek. Dereplication mode was set to one result per strain. The maximum gene cluster size was set to 60 kb, and flanking region size was set to 75 kb. Files with BGCs were exported in GenBank file format and used as input for BisCEET. To prepare the GenBank files from CluSeek prior to loading into BisCEET, empty SOURCE and ORGANISM fields in the GenBank files were populated with the species name present in the file name of the respective CluSeek output files.

## Supplementary Material



## Data Availability

The BisCEET source code, user documentation, and dereplicated BGCs from analyses showcased in this work (for use as test examples) are freely available at https://github.com/stsowa/BisCEET. The software is implemented in Python and released under the MIT license. The data not included within the article or the Supporting Information will be shared on reasonable request to the corresponding author.

## Notes

User interface of BisCEET as well as GenBank accession identifiers related to genome mining efforts (PDF)
