# Supplementary material for: BisCEET: A Visual Browser for Biosynthetic Gene Clusters Aiding in the Identification of Natural Product Variants and Distinct Tailoring Enzymes
Source: J Nat Prod. 2026 Apr 29;89(5):1392–8. doi: 10.1021/acs.jnatprod.5c01563 (PMC13200232; doi:10.1021/acs.jnatprod.5c01563)
Supplement: Supplementary file 1 [file np5c01563_si_001.pdf]

Supporting information

BisCEET: A visual browser for biosynthetic gene clusters aiding in the identification of natural product variants and distinct tailoring enzymes

Sven T. Sowa[1], Heiner G. Weddeling[1] and Robin Teufel[1*]

[1] Pharmaceutical Biology, Department of Pharmaceutical Sciences, University of Basel, Klingelbergstrasse 50, 4056 Basel (Switzerland).

*E-mail: robin.teufel@unibas.ch

Contents

Figure S1: Graphical interface and functionality of BisCEET. 2

Figure S2: Visual alignment of BGCs in BisCEET. 3

Figure S3: Coloring by gene and by groups. 4

Figure S4: Options to visually highlight potentially novel biosynthetic genes. 5

Table S1: Potential additional variant-specific tailoring genes in the staurosporine-related BGC from *Lentzea kristufekii*. 6

Table S2: Potential additional variant-specific tailoring genes in the staurosporine-related BGC from Frankia sp. Cas4. 6

Table S3: Potential additional variant-specific tailoring genes in the K-252a-related BGC from *Streptomyces* sp. MUM 2J. 6

Table S4: Potential additional variant-specific tailoring genes in the xantholipin-related BGC from *Streptomyces* sp. NBC_01538. 6

Table S5: Potential additional variant-specific tailoring genes in the xantholipin-related BGC from *Streptomyces* sp. CA-250714. 7

Table S6: Potential additional variant-specific tailoring genes in the lysolipin-related BGC from *Streptomyces* sp. 2323.1. 7

Table S7: Potential additional variant-specific tailoring genes in the lysolipin-related BGC from *Streptoverticillium reticulum*. 7

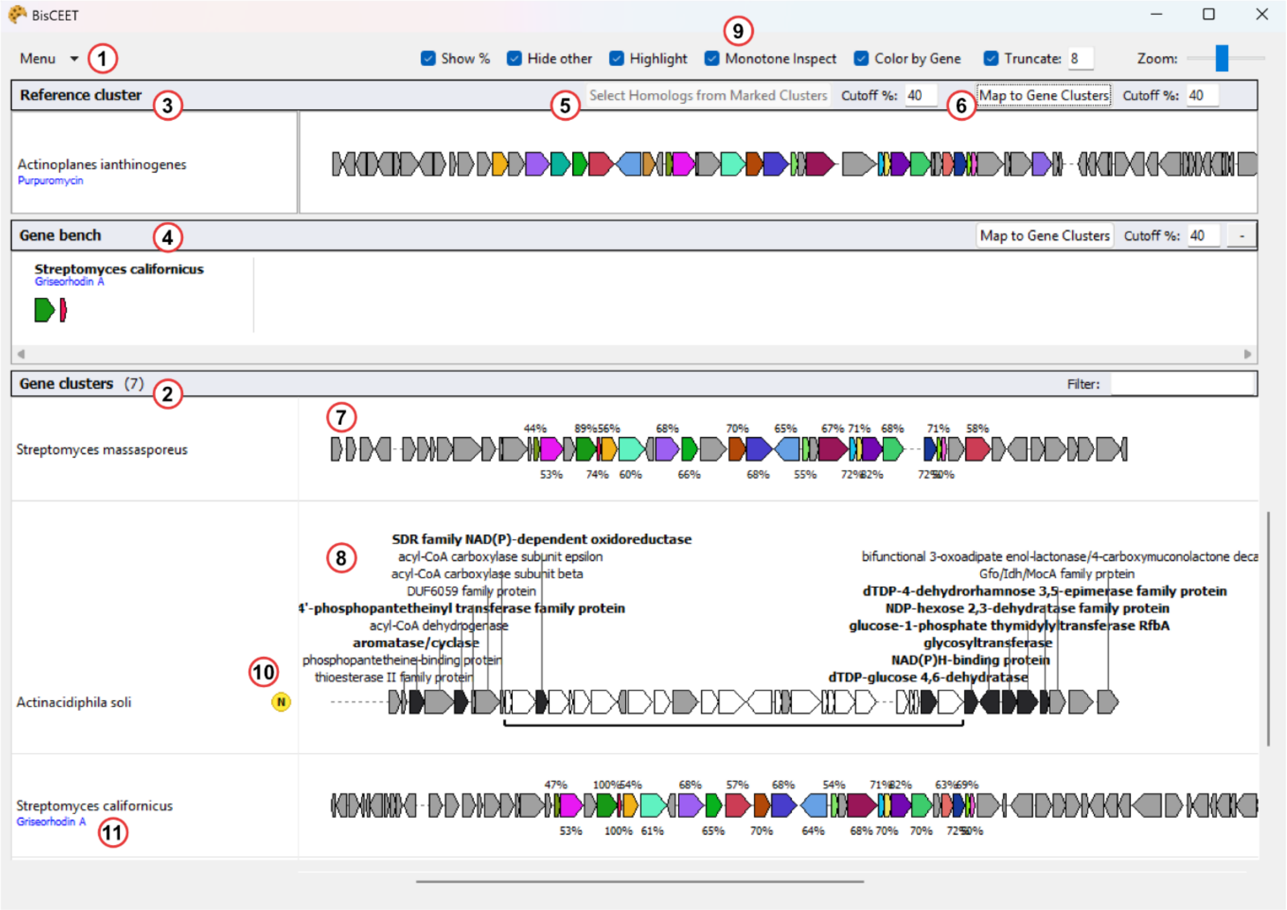

Figure S1: Graphical interface and functionality of BisCEET. (**1**) Load gene clusters in .gb or .gbk format or save and load BisCEET-project files. (**2**) Gene clusters appear in a list, initially genes are not colored. (**3**) A reference cluster from the list of BGCs is selected. (**4**) Individual genes from the list of BGCs or from user-defined sequences can be saved in the 'Gene bank' section. (**5**) Genes that have homologs in the reference BGC and other marked BGCs from the list will be automatically selected in the reference BGC. (**6**) Genes selected in the reference BGC and the Gene bench will be mapped (color coded) to the list of BGCs. (**7**) Mapped genes appear colored and the sequence identity of each gene to its homolog in the reference BGC is displayed. (**8**) A single or all BGCs from the list can be displayed in 'inspection view', showing labels for all non-mapped genes. (**9**) Multiple customization options are available for displaying the BGC list: Percentages of sequence identities to genes in the homolog can be shown. Annotations of genes considered not relevant (e.g. transporter, symporter, regulator) can be hidden. Genes with interesting annotations (e.g. cytochrome P450, transferase, epimerase) can be highlighted (shown in bold with genes as black arrows). BGCs in 'inspect view' can be shown in monotone white/grey/black colors to remove visual distractions. Coloring of the mapped genes can be displayed per gene or per cluster (reference and gene bench groups). (**10**) Annotations in the form of text notes can be added to any BGC. BGCs with notes appear with a yellow 'N' (=Note) symbol. These notes can be opened and edited. (**11**) The product of known BGCs can be added and is then displayed in the list.

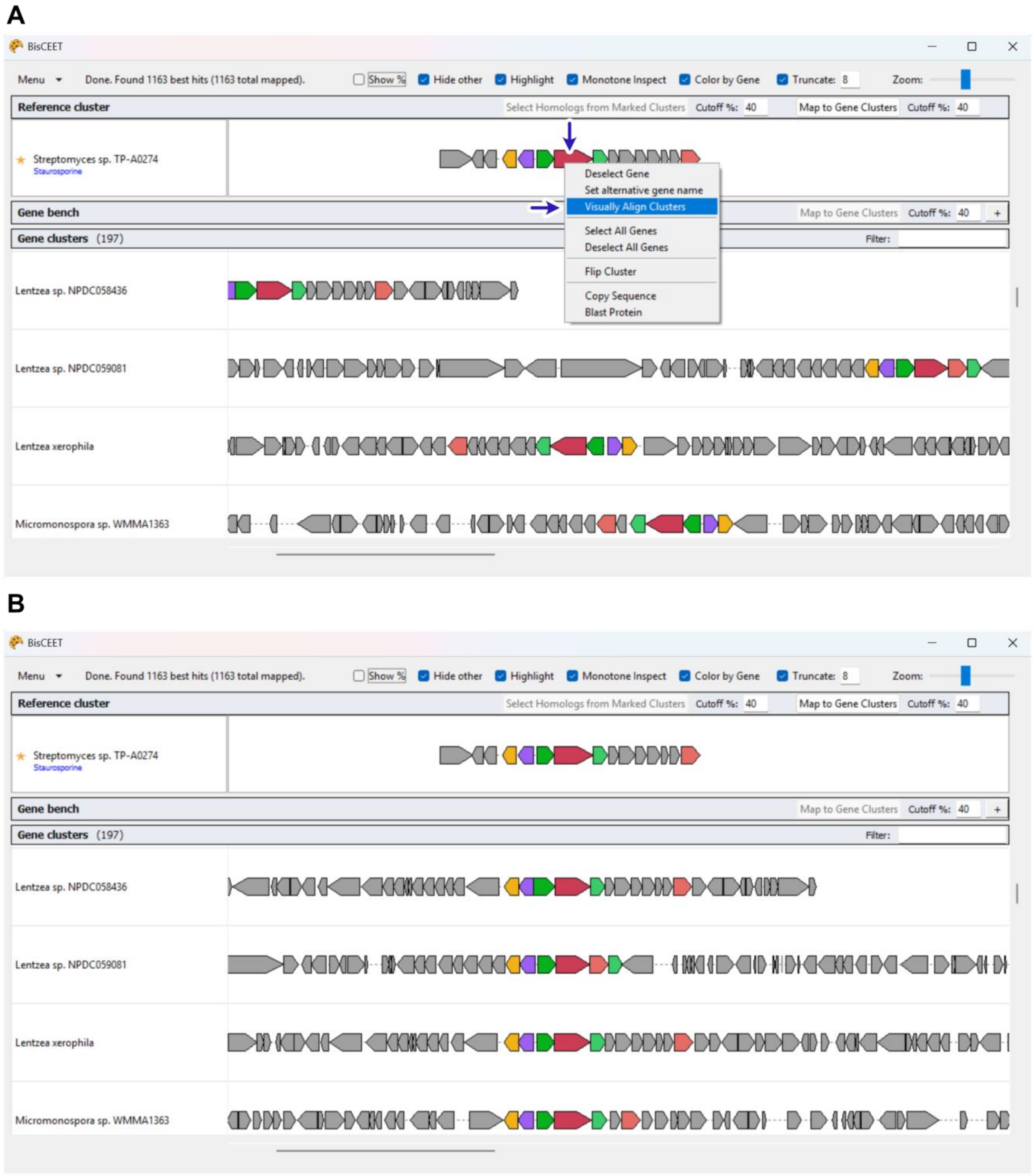

Figure S2: Visual alignment of BGCs in BisCEET. (**A**) BGCs are typically not aligned upon loading them into BisCEET due to varying sizes, completeness, orientation and gene composition. By selecting "Visually Align Clusters" on a gene from the reference cluster (indicated by blue arrows), all BGCs containing a mapped homolog of this gene will be arranged to visually be aligned with the selected gene. (**B**) Result of aligned BGCs. Both horizontal positions as well as BGC orientations are aligned with the selected gene.

A

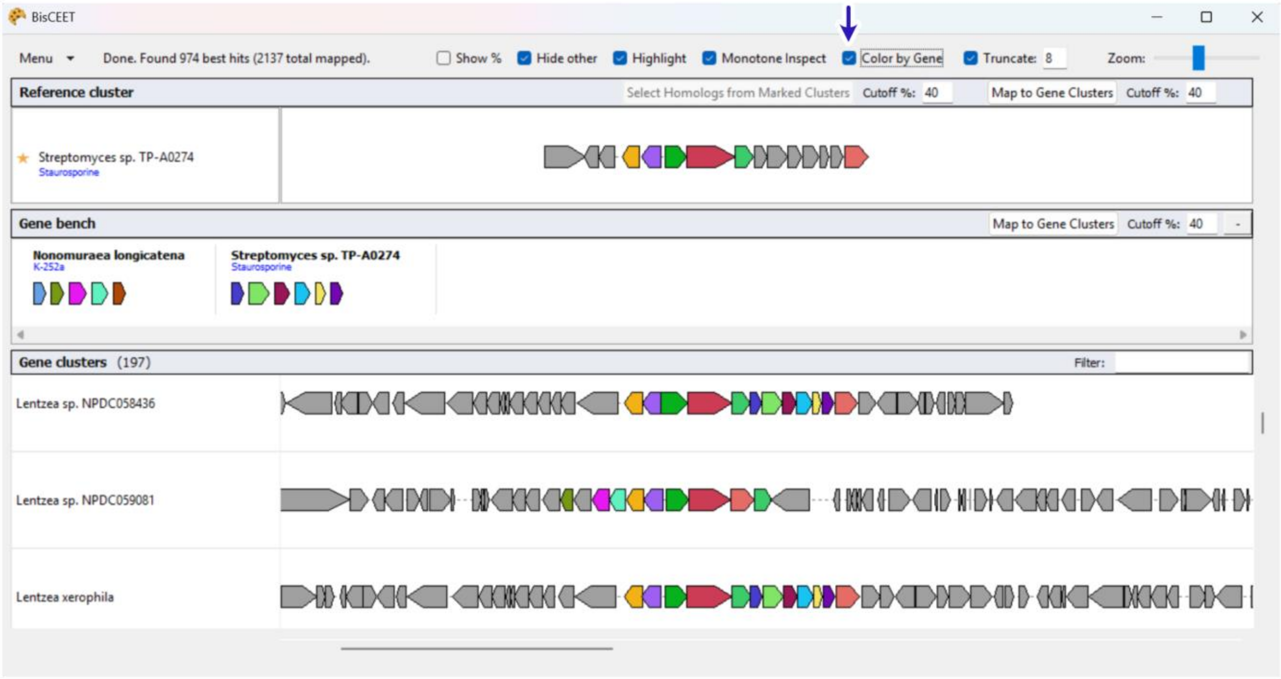

B

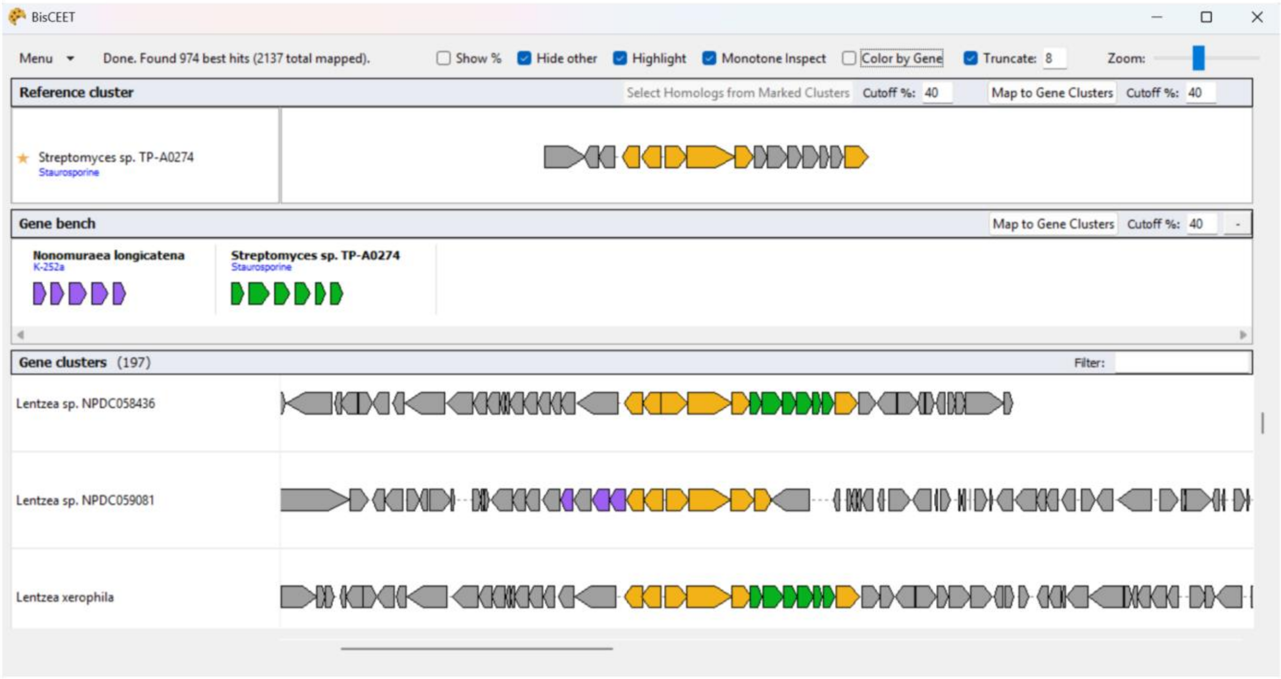

Figure S3: Coloring by gene and by groups. (**A**) By default, each gene selected in the reference cluster and the gene bench is colored individually, as determined by the activated “Color by Gene” option (indicated by the blue arrow). (**B**) By turning off the “Color by Gene” option, genes are colored based on groups. The reference cluster forms a group, and each set of genes pinned to the gene bench from the same species forms a group. In the example, the core biosynthetic genes are marked in the reference cluster (yellow). Additional biosynthetic genes from the biosynthesis of K-252a are colored violet, while those for staurosporine are colored green. Using this coloring scheme, it is much easier to identify BGCs in the list that likely belong (or are more closely related) to a certain congener based on the colored additional biosynthetic genes.

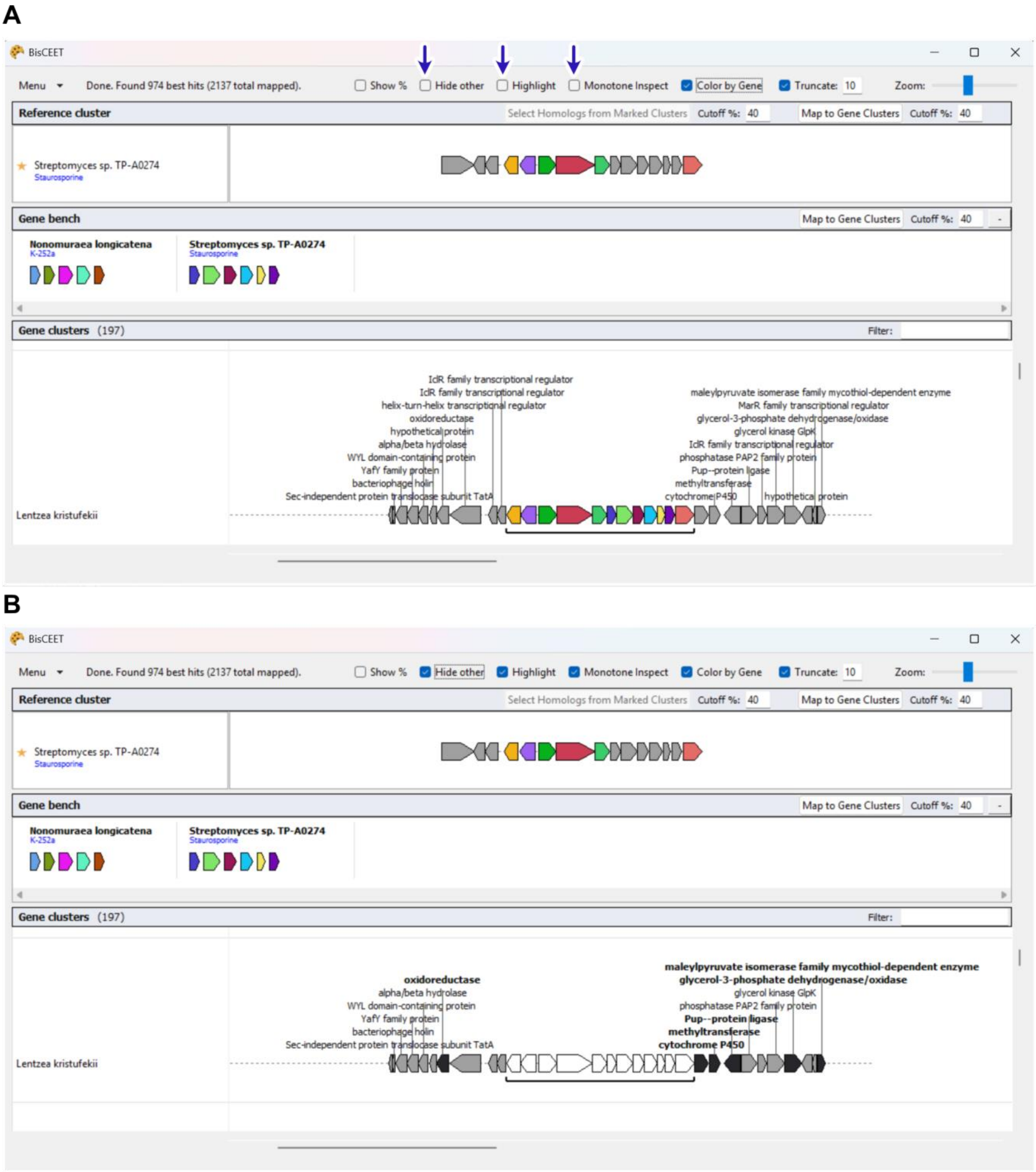

Figure S4: Options to visually highlight potentially novel biosynthetic genes. (**A**) A cluster from *Lentzea kristufekii* is shown in "Inspect View" with the "Hide other", "Highlight", and "Monotone Inspect" options toggled off (indicated by the blue arrows). For all non-mapped genes, annotations are displayed, and mapped genes appear colored. This makes it hard to visually focus on annotations of potentially biosynthetically important genes. (**B**) The same cluster in "Inspect View" with the "Hide other", "Highlight", and "Monotone Inspect" options toggled on, making it easier to visually focus on annotations of genes with biosynthetic relevance. "Hide other" option: Biosynthetically less relevant annotations containing e.g. "transporter", "regulator" or "symporter" are no longer displayed. "Highlight" option: Biosynthetically more relevant annotations containing e.g. "cytochrome P450", "methyltransferase" or "oxidoreductase" highlighted in bold with gene arrows displayed in black. "Monotone Inspect" option: Mapped genes are displayed in just white instead of color.

Table S1: Potential additional variant-specific tailoring genes in the staurosporine-related BGC from *Lentzea kristufekii*.

Position	GenBank identifier	Annotation
1	MDX8054396.1	cytochrome P450
2	MDX8054395.1	methyltransferase

Table S2: Potential additional variant-specific tailoring genes in the staurosporine-related BGC from Frankia sp. Cas4. WP_322778113.1 was annotated as "hypothetical protein", but homology searches suggest this may be a SAM-dependent methyltransferase.

Position	GenBank identifier	Annotation
1	WP_322778105.1	cytochrome P450
2	WP_322778106.1	cytochrome P450
3	WP_322778110.1	KedN5 family methylcobalamin-dependent radical-SAM C-methyltransferase
4	WP_322778111.1	SAM-dependent methyltransferase
5	WP_322778112.1	cytochrome P450
6	WP_322778113.1	hypothetical protein (SAM-dependent methyltransferase)
7	WP_322778114.1	cytochrome P450

Table S3: Potential additional variant-specific tailoring genes in the K-252a-related BGC from *Streptomyces* sp. MUM 2J.

Position	GenBank identifier	Annotation
1	MCH0566879.1	FAD-dependent monooxygenase
2	MCH0566864.1	FAD-binding oxidoreductase

Table S4: Potential additional variant-specific tailoring genes in the xantholipin-related BGC from *Streptomyces* sp. NBC_01538.

Position	GenBank identifier	Annotation
1	WTJ85791.1	multicopper oxidase domain-containing protein
2	WTJ85804.1	amino acid adenylation domain-containing protein
3	WTJ85805.1	condensation domain-containing protein
4	WTJ85806.1	cytochrome P450
5	WTJ85823.1	NAD(P)-dependent oxidoreductase

Table S5: Potential additional variant-specific tailoring genes in the xantholipin-related BGC from *Streptomyces* sp. CA-250714.

Position	GenBank identifier	Annotation
1	MGI5351671.1	NAD(P)-dependent oxidoreductase
2	MGI5351685.1	NAD(P)-dependent oxidoreductase
3	MGI5351694.1	multicopper oxidase family protein
4	MGI5351698.1	cytochrome P450
5	MGI5351701.1	acyl-CoA dehydrogenase family protein
6	MGI5351703.1	bifunctional 2-polyprenyl-6-hydroxyphenol methylase / 3-demethylubiquinol 3-O-methyltransferase UbiG
7	MGI5351705.1	type III polyketide synthase
8	MGI5351706.1	aminotransferase class III-fold pyridoxal phosphate-dependent enzyme
9	MGI5351707.1	aminotransferase class III-fold pyridoxal phosphate-dependent enzyme
10	MGI5351708.1	AMP-binding protein
11	MGI5351709.1	NADPH-dependent FMN reductase
12	MGI5351710.1	tryptophan-synthase subunit alpha
13	MGI5351711.1	methyltransferase-domain containing protein
14	MGI5351725.1	multicopper oxidase family protein
15	MGI5351730.1	NAD(P)/FAD-dependent oxidoreductase

Table S6: Potential additional variant-specific tailoring genes in the lysolipin-related BGC from *Streptomyces* sp. 2323.1.

Position	GenBank identifier	Annotation
1	SOE10360.1	protein-L-isoaspartate(D-aspartate) O-methyltransferase
2	SOE10359.1	Transglutaminase-like superfamily protein
3	SOE10358.1	Coenzyme PQQ synthesis protein D (PqqD)
4	SOE10357.1	asparagine synthase (glutamine-hydrolyzing)

Table S7: Potential additional variant-specific tailoring genes in the lysolipin-related BGC from *Streptoverticillium reticulum*.

Position	GenBank identifier	Annotation
1	XOA49518.1	dTDP-4-dehydrorhamnose reductase
2	XOA49517.1	dTDP-4-dehydrorhamnose 3,5-epimerase
3	XOA50490.1	glucose-1-phosphate thymidylyltransferase RfbA
4	XOA49511.1	glycosyltransferase
5	XOA49493.1	class I SAM-dependent methyltransferase
6	XOA49479.1	NDP-hexose 2,3-dehydratase family protein
7	XOA49478.1	glycosyltransferase
8	XOA49477.1	TylF/MycF family methyltransferase
